# Partial Recovery of Telomere Length After Long-term Virologic Suppression in Persons With HIV-1

**DOI:** 10.1093/ofid/ofae550

**Published:** 2024-09-23

**Authors:** Julen Cadiñanos, Javier Rodríguez-Centeno, Rocío Montejano, Andrés Esteban-Cantos, Beatriz Mena-Garay, María Jiménez-González, Gabriel Saiz-Medrano, Rosa de Miguel, Fernando Rodríguez-Artalejo, José I Bernardino, Cristina Marcelo-Calvo, Lucía Gutierrez-García, Patricia Martínez-Martín, Alejandro Díez Vidal, Alejandro de Gea Grela, Rosario Ortolá, Berta Rodés, José R Arribas

**Affiliations:** Department of Internal Medicine, La Paz University Hospital–IdiPAZ, Madrid, Spain; CIBER of Infectious Diseases, Madrid, Spain; Department of Internal Medicine, La Paz University Hospital–IdiPAZ, Madrid, Spain; CIBER of Infectious Diseases, Madrid, Spain; Department of Internal Medicine, La Paz University Hospital–IdiPAZ, Madrid, Spain; CIBER of Infectious Diseases, Madrid, Spain; Department of Internal Medicine, La Paz University Hospital–IdiPAZ, Madrid, Spain; CIBER of Infectious Diseases, Madrid, Spain; Department of Internal Medicine, La Paz University Hospital–IdiPAZ, Madrid, Spain; Department of Internal Medicine, La Paz University Hospital–IdiPAZ, Madrid, Spain; Department of Internal Medicine, La Paz University Hospital–IdiPAZ, Madrid, Spain; Department of Internal Medicine, La Paz University Hospital–IdiPAZ, Madrid, Spain; CIBER of Infectious Diseases, Madrid, Spain; Department of Preventive Medicine and Public Health, Universidad Autónoma de Madrid, Madrid, Spain; CIBER of Epidemiology and Public Health, Madrid, Spain; IMDEA–Food Institute, Madrid, Spain; Department of Internal Medicine, La Paz University Hospital–IdiPAZ, Madrid, Spain; CIBER of Infectious Diseases, Madrid, Spain; Department of Internal Medicine, La Paz University Hospital–IdiPAZ, Madrid, Spain; Department of Internal Medicine, La Paz University Hospital–IdiPAZ, Madrid, Spain; Department of Internal Medicine, La Paz University Hospital–IdiPAZ, Madrid, Spain; Department of Internal Medicine, La Paz University Hospital–IdiPAZ, Madrid, Spain; CIBER of Infectious Diseases, Madrid, Spain; Department of Internal Medicine, La Paz University Hospital–IdiPAZ, Madrid, Spain; Department of Preventive Medicine and Public Health, Universidad Autónoma de Madrid, Madrid, Spain; CIBER of Epidemiology and Public Health, Madrid, Spain; Department of Internal Medicine, La Paz University Hospital–IdiPAZ, Madrid, Spain; CIBER of Infectious Diseases, Madrid, Spain; Department of Internal Medicine, La Paz University Hospital–IdiPAZ, Madrid, Spain; CIBER of Infectious Diseases, Madrid, Spain

**Keywords:** aging, HIV, immune reconstitution, telomere

## Abstract

**Background:**

People with HIV-1 (PWH) age differently than the general population. Blood telomere length (BTL) attrition is a surrogate biomarker of immunosenescence and aging in PWH. BTL is reduced immediately after HIV-1 infection and recovers in PWH with long-term virologic suppression, but the extent of this recovery is unknown.

**Methods:**

This prospective 6-year observational study assessed the evolution of BTL in PWH who were virologically suppressed. A cross-sectional analysis additionally compared BTL with age- and sex-matched blood donors and sex-matched persons older than 60 years from a general population cohort. DNA from whole blood was isolated, and relative BTL was determined by monochrome quantitative multiplex polymerase chain reaction assay and expressed as the ratio of telomere to single-copy gene (T/S).

**Results:**

A total of 128 PWH were included in the prospective 6-year observational study. These same 128 PWH (median age, 55 years; 27.3% women) were compared cross-sectionally at 6-year follow-up with 128 age- and gender-matched blood donors (median age, 55 years) and 128 gender-matched individuals older than 60 years from a general population cohort (median age, 70 years). An inverse correlation between age and BTL was observed. The median BTL of PWH was shorter than their matched blood donors (T/S, 1.07 [IQR, 0.95–1.17] vs 1.28 [IQR, 1.12–1.48]; *P* < .001) but longer than the elderly population (T/S, 0.89 [IQR, 0.77–0.98], *P* < .001). PWH experienced a BTL increase at 6 years of 2.9% (T/S, 1.04 vs 1.07; *P* = .002). In PWH, age was associated with a shorter BTL (coefficient, −0.007 45, SE = 0.002 04, *P* = .002) and baseline lower CD4 count with a gain in BTL (coefficient, −0.000 06, SE = 0.000 02, *P* = .004). Shorter baseline BTL (odds ratio, 0.91 [95% CI, .87–.94]; *P* < .001) and higher glucose levels (odds ratio, 1.04 [95% CI, 1.02–1.07]; *P* = .003) were associated with a greater similarity of BTL to the elderly population.

**Conclusions:**

PWH with long-term virologic suppression experience a trend toward an increased BTL after 6 years of follow-up. Middle-aged people with long-term controlled HIV-1 have a shorter BTL than expected for their chronologic age but longer than that of people 15 years older in the general population.

Antiretroviral therapy (ART) has drastically changed the prognosis of HIV-1 infection, from being a frequently fatal illness to a chronic manageable disease. As a result, people with HIV-1 (PWH) are aging, and their life expectancy has been approaching that of the general population [1]. An overall increased risk of age-related comorbidities has been described in PWH as compared with the general population of the same age [2], which suggests that PWH still age differently from the general population and have either accelerated aging (age-related illnesses that occur earlier than expected) or accentuated aging (more frequently than expected) [3].

Studying the factors promoting accelerated or accentuated aging in chronic diseases is a challenge given the need for a large sample and a prolonged follow-up to find a significant impact of these factors in mortality or aging-related diseases. Aging biomarkers as a proxy end point are an attractive alternative. Blood telomere length (BTL) is a well-known biomarker of cellular aging [4], correlates with age [5], and has been associated with mortality and aging-related diseases in the general population and in PWH [6, 7].

Telomere shortening in peripheral blood mononuclear cells occurs shortly after HIV-1 seroconversion [8]. When compared with individuals matched by age who were uninfected, PWH have a shorter telomere length [9], especially untreated individuals [10]. We and others have shown that initiation of ART in PWH is followed by an increase in BTL [11]. However, the dynamics and factors involved in BTL after long-term virologic suppression are not well described. The mechanisms by which HIV-1 infection could affect immunosenescence and specifically telomere length are likely complex: chronic activation and exhaustion of the immune system are involved, and a direct telomerase inhibition by viral proteins has been described [12]. Despite the overall benefit of ART, a negative impact of antiretroviral drugs on BTL cannot be ruled out [13]. Comorbidities and lifestyle habits that can affect BTL and are prevalent in this population make it difficult to single out the direct effect of HIV-1 infection.

Given the uncertainties surrounding BTL in PWH following long-term virologic suppression, our study aims to evaluate the recovery of BTL in PWH with long-term virologically suppressed infection after 6 years of follow-up and to compare their BTL with that of people without HIV-1 infection of the same and older chronologic age. We hypothesize that after an additional 6 years of virologic suppression, BTL in PWH will show signs of improvement or stabilization yet remain shorter than that of their age- and sex-matched controls without HIV-1 infection, resembling more closely the BTL of an older population.

## METHODS

### Study Design and Population

Our study includes a prospective observational cohort evaluating the evolution of BTL in PWH under virologic suppression and a cross-sectional analysis to compare the BTL of PWH with that of individuals without HIV-1 infection.

The prospective observational cohort included 128 PWH with long-term virologically suppressed infection. Details of this ongoing cohort have been described [14]. Enrollment took place at La Paz University Hospital (Madrid, Spain) between March 2014 and March 2015 and involved PWH on stable ART regimen who were virologically suppressed for at least 12 months. Two visits were performed to collect clinical information and draw blood (baseline and after 6 years of follow-up). We reviewed medical records from the PWH group to retrieve clinical information. Participants were interviewed on the day of the visit to collect other relevant information related to BTL.

For the cross-sectional study, we compared the BTL of the PWH group (baseline and at 6 years of follow-up) with single-draw whole blood samples from 2 populations: elderly persons from the general population and blood donors. Blood donors’ samples were obtained from the Transfusion Center of the Community of Madrid, which provided remnant blood samples from 128 healthy donors matched with the PWH group by sex and age at 6 years of follow-up. The elderly population group included blood samples from the Seniors-ENRICA-1 cohort (ClinicalTrials.gov NCT01133093) [15], a noninstitutionalized general population aged >60 years who were randomly recruited from the Spanish population between 2008 and 2010 to carry out studies on nutrition and cardiovascular risk. We selected 128 individuals of this cohort, with the same sex distribution and a median age approximately 15 years older than the PWH group at 6 years of follow-up.

### Ethics Statement

The study protocol was approved by the Clinical Research Ethics Committee of La Paz University Hospital (Madrid, Spain; code PI-4007). Written informed consent was obtained from all participants.

### Laboratory Procedures

Genomic DNA from blood samples was purified with the QIAamp DNA Blood Kit (QIAGEN). Relative telomere length, expressed as the ratio of telomere to single-copy gene (T/S), was determined by monochrome quantitative multiplex polymerase chain reaction assay, with minor modifications as described in our prior study [14]. A standard curve was performed with genomic DNA from Human Tumor Cell Line K562 (BioChain) by serial dilution and was included in triplicate in each run with a reference sample and a negative control. Samples were randomized and assayed in triplicate, and those with a coefficient of variation >10% were reanalyzed.

### Statistical Analysis

A descriptive analysis was performed on all clinical and analytic variables. Characteristics of the participants were described by absolute and relative frequencies for qualitative variables and medians and IQR for quantitative variables. The normality of the variables was studied with the Kolmogorov-Smirnov normality test. The Mann-Whitney *U* test and Kruskal-Wallis test were used for quantitative variables. For qualitative variables, the chi-square test was used or Fisher exact test when necessary. For the correlation between BTL and the age of all groups, the Spearman correlation coefficient was used. To evaluate the variables associated with having a BTL similar to that of blood donors and the elderly population group, we assessed those PWH who had a better or similar BTL than their age- and sex-matched blood donors (within an established range of ±10%) and similar or worse BTL than the median BTL of the elderly population group (within an established range of ±10%). To look for an association between the study groups, multivariate logistic regression was fitted with the variables that were significant in the univariate analysis. The search for the best model was performed via the backward method, and the selection of the optimal model was performed via the Bayesian information criterion method.

## RESULTS

We included 128 participants, 27.3% female, in each of the 3 groups. The elderly population group was a median 15 years older than PWH and blood donors (median age, 70 vs 55 years at 6 years of follow-up). The main characteristics of the PWH group are described in Table 1. During the 6 years of follow-up, participants continued their routine clinic visits every 6 to 12 months, including the study visits, without any virologic failure being observed.

**Table 1.** Participant Characteristics of the HIV-1 Group After 6 Years of Follow-up (N = 128)

	No. (%) or Median (IQR)
Age, y	55 (51–58.2)
Female sex at birth	35 (27.3)
Smoking	
Smoker	59 (46.1)
Years	36 (29–41)
Pack-year	20 (10–39)
Former	41 (32)
Years	29 (20–39)
Pack-year	20 (12–38)
Never	28 (21.9)
Alcohol^a^	
Current hazardous alcohol consumption	7 (5.5)
Years	40 (35–43)
Standard drink units	28 (28–35)
Former	2 (1.6)
Nonsignificant alcohol consumption	119 (93)
Persons who inject drugs	
Current	0 (0)
Ever	41 (32)
Body mass index, kg/m	24.7 (22.4–27.9)
Ethnicity	
Caucasian	118 (92.2)
Other	10 (7.8)
Income^b^	
Lower: ≤12 000€/y	50 (39.1)
Higher: >12 000€/y	78 (60.9)
Education	
Primary	52 (40.6)
Secondary	40 (31.3)
University	36 (28.1)
Comorbidities	
None	75 (58.6)
1	29 (22.7)
≥2	24 (18.8)
Hypertension	43 (33.6)
Diabetes mellitus	24 (18.8)
Treatment with statins	42 (32.8)
Chronic kidney disease	6 (4.9)
Coronary artery disease	4 (3.1)
Peripheral artery disease	2 (1.6)
HCV coinfection	
No	82 (64.1)
Resolved	44 (34.4)
Active	2 (1.6)
Positive CMV serology (immunoglobulin G)	116 (90.6)
HIV-1 transmission route	
Sexual	83 (64.8)
Parenteral	41 (32)
Unknown	4 (3.2)
Previous AIDS	72 (56.2)
Known time with HIV-1 infection, y	23.6 (18.5–27.9)
Time virologically suppressed, y	13.2 (12.1–13.7)
Nadir CD4 count, cells/µL	181.5 (80.5–255)
ART regimen	
Triple therapy	74 (57.8)
NRTI backbone	
TDF/FTC	11 (8.6)
TAF/FTC	35 (27.3)
ABC/3TC	28 (21.9)
Other combinations of 2 NRTIs	0 (0)
Boosted PI monotherapy	10 (7.8)
Other NRTI-sparing regimen	44 (34.4)
Treatment with TDF/TAF	
Current	46 (35.9)
Time with TDF/TAF, y	16.1 (14.7–17.2)
Ever	64 (50)
Time with TDF/TAF, y	15.5 (11.5–16.9)
Never	64 (50)
Treatment with ABC	
Current	28 (21.9)
Time with ABC, y	9.9 (7.7–11.5)
Ever	76 (59.4)
Time with ABC, y	9.83 (5.1–12.9)
Never	52 (40.6)
Treatment with TDF/TAF or ABC	
Current or ever	120 (93.8)
Time with TDF/TAF or ABC, y	14.33 (10–16.8)
Never	8 (6.2)
Laboratory values ​	
Plasma HIV-1 load, copies/mL	0 (0–0)
CD4 count, cells/µL	773 (540–1020)
CD8 count, cells/µL	674 (470–930)
CD4/CD8 ratio	1.1 (0.8–1.4)
Glucose, mg/dL	91 (82.8–101)
Creatinine, mg/dL	0.85 (0.75–0.93)
C-reactive protein, mg/L	1.3 (0–4.3)
Cholesterol, mg/dL	
Total	181 (152–204.3)
HDL	48.5 (39–58)
LDL	108 (85–131)
Triglycerides, mg/dL	97.5 (75.6–151.3)
Fibrinogen, mg/dL	308 (206–372.8)
D-dimer, ng/mL	220 (170–313)

Abbreviations: 3TC, lamivudine; ABC, abacavir; ART, antiretroviral therapy; CMV, cytomegalovirus; FTC, emtricitabine; HCV, hepatitis C virus; HDL, high-density lipoprotein; LDL, low-density lipoprotein; NRTI, nucleoside/nucleotide reverse transcriptase inhibitor; PI, protease inhibitor; TAF, tenofovir alafenamide; TDF, tenofovir disoproxil fumarate.

^a^Hazardous alcohol consumption was defined as consuming >28 or >17 standard drinking units/wk in men and women, respectively.

^b^12 000€/y is the average net income per person in Spain in 2020.

**Figure 1.**
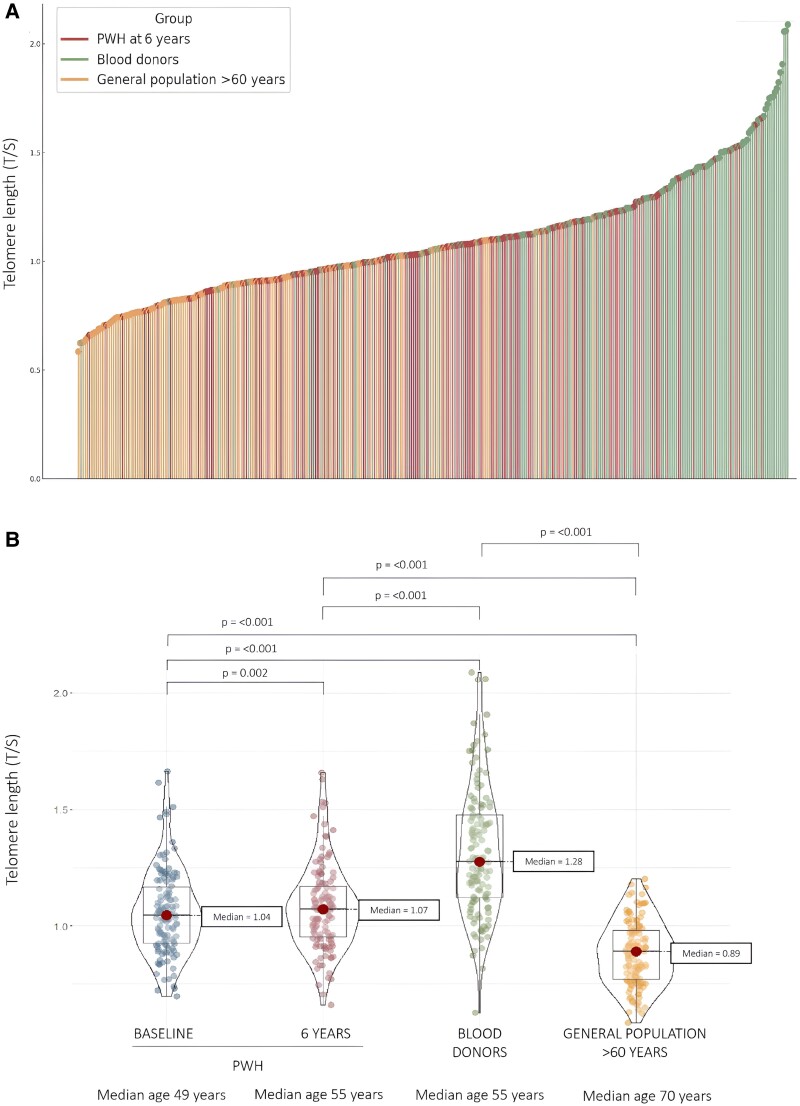
Differences in blood telomere length (BTL) among the groups. *A*, This lollipop diagram shows an individual BTL, measured as the ratio of telomere to single-copy gene (T/S), for each participant in ascending order: persons with HIV-1 (PWH) after a 6-year follow-up (pink dots), age/sex-matched blood donors (green dots), and the general population aged >60 years (orange dots). *B*, This violin plot compares the BTL in the 3 groups of participants. Dots represent individual data: PWH at baseline (blue) and after a 6-year follow-up (pink), age/sex-matched blood donors (dots), and the general population aged >60 years (orange). Red dots show the medians and box plots show IQRs. The width of each curve corresponds to the approximate frequency of data points for a given BTL.

**Figure 2.**
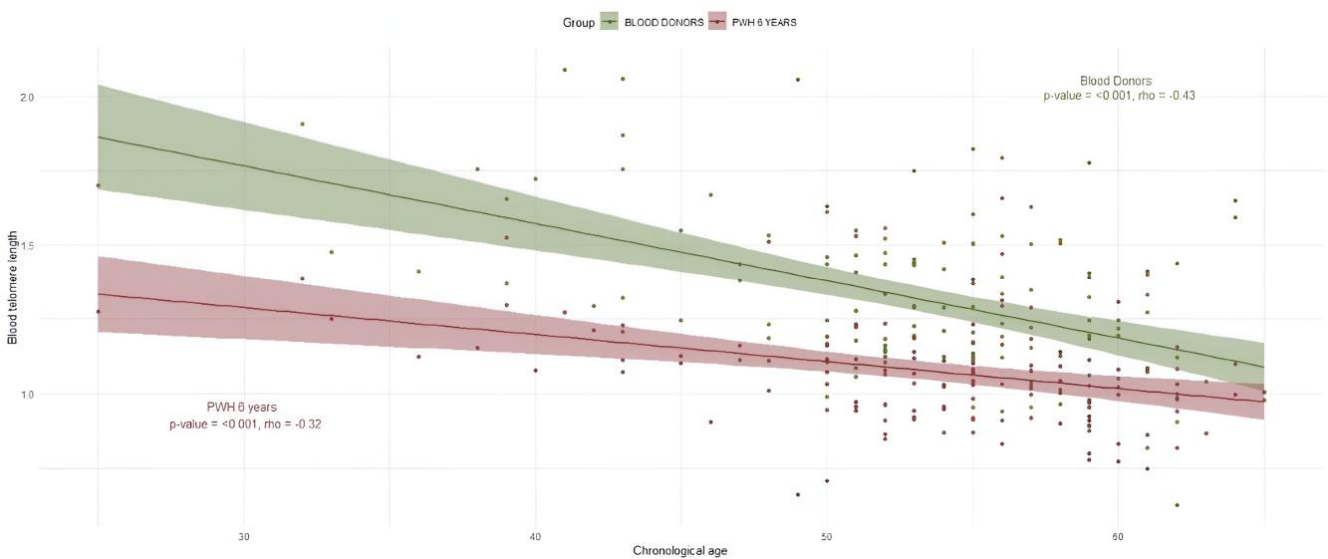
Correlation between age and blood telomere length in people with HIV-1 (PWH) and blood donors at 6 years of follow-up. Dots show individual data, and lines show correlation curves of PWH (red) and their age- and sex-matched blood donors (green). Shaded areas represent the 95% CI.

PWH had a significantly shorter median BTL than the age- and sex-matched blood donor (T/S, 1.07 [IQR, 0.95–1.17] vs 1.28 [IQR, 1.12–1.48]; *P* < .001) but one significantly above the median BTL of the elderly population group (0.89 [IQR, 0.77–0.98], *P* < .001; Figure 1). An inverse correlation between age and BTL was observed (Figure 2, Supplementary Figure 1).

Globally, the median BTL of the PWH group increased 2.9% after 6 years (+0.48% per year; T/S, 1.04 [IQR, 0.93–1.16] at baseline vs 1.07 [IQR, 0.95–1.17] at 6 years of follow-up; *P* = .002; Supplementary Figure 2), and 58.4% of participants had a longer BTL at the end of follow-up than at baseline. We evaluated the possible factors associated with BTL (T/S at 6 years) and with the change of BTL after 6 years of follow-up. Only age was significantly associated with a shorter BTL (coefficient, −0.007 45, SE = 0.002 04, *P* = .002; Supplementary Table 1). Lower baseline CD4 count was the only variable associated with BTL gain (coefficient, −0.000 06, SE = 0.000 02, *P* = .004; Supplementary Table 2). We found no association with ART regimen, including previous or current use, or time of exposure to tenofovir disoproxil fumarate/tenofovir alafenamide (TDF/TAF), abacavir (ABC), or both.

We evaluated the variables of the PWH group associated with the similarity of BTL to healthy donors or the elderly population. In total, 78 (60.9%) PWH had a shorter BTL than their age- and sex-matched blood donors. We analyzed the potential clinical and analytic age-related variables associated with having a better or similar BTL than blood donors. In the univariate analysis, current or previous treatment with TDF/TAF or ABC, higher glucose levels, and baseline BTL were associated with similarity of BTL to healthy donors, but no association was found in the multivariate analysis (Supplementary Figure 3, Supplementary Table 3). An overall 42 (32.8%) PWH had a similar or worse BTL than the median of the elderly population. In the multivariate analysis, shorter baseline BTL (odds ratio, 0.91; 95% CI, .87–.94; *P* < .001) and higher glucose levels (odds ratio, 1.04; 95% CI, 1.02–1.07; *P* = .003) were associated with a greater similarity of PWH to the elderly population group (Supplementary Figure 4, Supplementary Table 4).

## DISCUSSION

Our findings indicate that PWH with long-term virologic suppression continue to experience gains in BTL after more than 1 decade of virologic suppression. Despite this prolonged immune reconstitution process, PWH have a shorter BTL than expected for their chronologic age, though their BTL is longer than that observed in older individuals from the general population. Overall, our study results underscore that even effectively treated HIV-1 infection exerts a detrimental effect on biological aging, an impact only partially mitigated by ART. To our knowledge, this is the largest study evaluating the evolution of BTL after long-term virologic suppression and the first study assessing the differences in BTL in PWH with long-term virologic suppression as compared with age- and sex-matched healthy controls and an elderly population.

This gain of BTL after the start of ART in PWH has been observed by our group and in other longitudinal studies [16, 17]. In the longest longitudinal study, including 107 PWH, Schoepf et al found rapid deterioration of telomere length in peripheral blood mononuclear cells during untreated infection (2.12% per year; median follow-up, 7.7 years) but no significant changes after initiation of ART (median follow-up, 9.8 years) [10]. There are several reasons that may explain the differences with our findings. Our study population is older, and the rate of BTL attrition at advanced ages tends to slow or stabilize [5]. Additionally, our larger sample size provides more statistical power to detect significant differences in the evolution of BTL. The follow-up period of the study by Schoepf et al is longer, and the potential beneficial impact of maintaining virologic suppression with ART may have a limit, beyond which age becomes more significant. A recent meta-analysis showed a greater decrease in telomere length over time in peripheral blood mononuclear cells when compared with other samples, such as whole blood [5]. Finally, we cannot rule out significant differences among populations in other characteristics that may influence BTL. In a smaller study including 31 persons who inject drugs, there was a significant decrease in BTL after seroconversion, but there were no significant differences during subsequent follow-up for a mean 2.2 years, although in this case just 22% of participants were receiving ART at the time of the last visit [18]. The findings of our study and the other longitudinal studies discussed here support that ART would have a beneficial effect on BTL and suggest that immune reconstitution persists beyond a decade of virologic suppression. ART would lead to a restoration of less mature T-cell phenotypes with a longer BTL, which could mitigate the expected shortening with age. Although our PWH cohort presented an overall gain in BTL, this did not occur in all participants, and we have not been able to find which factors predict the possibility of gaining BTL.

We found a good correlation between age and BTL in PWH and blood donors. According to the correlation curves between age and BTL of PWH and blood donors, we observed a difference corresponding to at least 10 years of accentuated BTL attrition in PWH, even at ages >50 years, as compared with blood donors. In a cross-sectional study, Zanet et al compared the BTL of PWH and people without HIV-1 infection and estimated premature aging of approximately 9 years for PWH [19]. In our study, the BTL curves of PWH and blood donors decrease with age almost in parallel, tending to converge just at older ages, probably due to the appearance of other factors associated with aging. In the study by Liu et al, PWH had a shorter BTL when compared with the general population of the same age (measured in absolute values, approximately 27 kbp/genome), and the correlation between age and BTL decreased in parallel in both groups [20]. The shorter BTL in PWH vs the general population, which remains stable throughout adulthood, would support the concept of accentuated rather than accelerated aging, in which we would have expected a progressive increase in the difference in BTL between PWH and the general population with age.

We have not found relevant factors associated with BTL or its change during follow-up in PWH. In our study, the only variable associated with BTL in PWH was age. Baseline CD4 count was the only variable associated with a BTL gain during follow-up, probably due to a greater potential for recovery in patients with a worse baseline immunologic condition. Shorter baseline BTL and higher glucose levels of PWH were the only variables associated with similarity to the elderly population. Since BTL shortens with age, it is logical that PWH with a shorter baseline BTL were more likely to have a BTL similar to the elderly population. In the general population, the prevalence of prediabetes and diabetes mellitus increases with age [21], and shorter BTL has been associated with the development of diabetes mellitus and with diabetes-related complications and mortality [22]. Hyperglycemia and diabetes mellitus are correlated with biological aging [23] and are a well-known risk factors for mortality in PWH [24].

We found no association between other common HIV-1 infection or age-related variables and BTL, BTL change during follow-up, or similitude of BTL to that of healthy donors or elderly controls. In a recent longitudinal study spanning >17 years with PWH, before and after the initiation of ART, our group found that maintaining virologic suppression was the most significant variable associated with epigenetic age deceleration. This underscores the critical importance of minimizing the duration of untreated HIV-1 infection [25]. Furthermore, in this study we did not find a relationship between epigenetic age acceleration and other potential aging-associated variables. This suggests that achieving virologic suppression exerts a more profound impact on BTL than other clinical, immunologic, and HIV-1–related factors.

In another cohort of participants with and without HIV-1 infection, smoking was associated with a shorter BTL in only the HIV-1–negative population [19]. In the study by Schoepf et al, no factors were associated with changes in telomere length in peripheral blood mononuclear cells from PWH with long-term virologic suppression [10]. We found a strong correlation between baseline BTL and BTL at 6 years of follow-up (Supplementary Figure 5), suggesting negligible differences in BTL recovery among PWH. Taken together, the evidence suggests that much of the BTL damage caused by HIV-1 infection occurs before achieving virologic control and that virologic suppression may have a major role in the recovery of BTL. This could account for the minimal influence of other potential age-related variables observed in our study and in previous research. We hypothesize that early initiation of ART and maintenance of virologic suppression are the key factors in mitigating aging processes in PWH, regardless of other factors, including the specific ART regimen.

Our study found no significant link between ART usage (including duration and specific drugs, such as TDF/TAF and ABC) and BTL, its changes over time, or similarities with control groups. The effect of ART on BTL is unclear [14, 20]: while some in vitro studies suggest that nucleoside/nucleotide reverse transcriptase inhibitors such as TDF and ABC could damage telomere integrity [13], our earlier research indicated a potential association between these drugs and reduced BTL gains [11], a finding not replicated in this smaller, longer-term study.

Our study has several limitations. It is an observational study, so biases cannot be excluded and we cannot rule out an influence of ART and other factors on BTL. Despite efforts to mitigate cohort study biases by comparing with 2 groups not infected with HIV-1, the lack of longitudinal data from these controls limits our understanding of BTL's natural progression. Only data on age and sex were available for these groups, omitting other factors that could affect BTL. Despite attempts to account for major BTL influencers in the HIV-1 group, unmeasured confounders such as diet and lifestyle might exist. We measured telomere length in whole blood samples, but the different lymphocyte subpopulations have differences in telomere length, and there might be relevant changes in lymphocyte subpopulations under effective ART. Other studies highlighted the importance of measuring the telomere length in the different lymphocyte subpopulations [26].

## CONCLUSION

PWH with long-term virologic suppression experience a trend toward increased BTL after 6 years of follow-up. Among middle-aged PWH who have achieved sustained viral suppression, BTL is shorter than what is typically expected for their age yet longer than that observed in the general elderly population >60 years old. This finding underscores the critical role of prolonged virologic suppression in influencing BTL dynamics in PWH, as it can slow down or potentially reverse the age-associated decline in BTL. The effectiveness of virologic suppression is such that it probably reduces the impact of other aging-related factors on BTL.

## Supplementary Data

Supplementary materials are available at *Open Forum Infectious Diseases* online. Consisting of data provided by the authors to benefit the reader, the posted materials are not copyedited and are the sole responsibility of the authors, so questions or comments should be addressed to the corresponding author.

## Supplementary Material

ofae550_Supplementary_Data

## Notes

***Acknowledgments.*** We are grateful to all study participants and their partners, families, and caregivers.

***Author contributors.*** J. C., J. R.-C., R. M., A. E.-C., B. R., and J. R. A. participated in the conceptualization and design of the study. J. C., R. M., and R. M. were study investigators and participated in the conduct of the study, including the recruitment and follow-up of participants. J. R.-C., A. E.-C., B. M.-G., and G. S.-M. performed the laboratory procedures. J. C., J. R.-C., A. E.-C., and M. J.-G. were involved with formal data analysis. B. R. and J. R. A. were responsible for funding acquisition and supervision of all the processes of the trial. All authors contributed to the interpretation of the results and participated in the drafting and review of the manuscript.

***Data sharing.*** Unidentified participant data and study documents will be accessible with a signed data access agreement upon approval of a proposal by email with the corresponding author, upon publication of the article.

***Financial support***. This work was supported by the Instituto de Salud Carlos III for Strategic Action in Health 2019 (PI19/00294); a Rio Hortega fellowship from Instituto de Salud Carlos III (CM19/00059 to J. C.); and a Sara Borrell fellowship from Instituto de Salud Carlos III (CD21/00051 to J. R.-C.).

## References

[1] Autenrieth CS, Beck EJ, Stelzle D, Mallouris C, Mahy M, Ghys P. Global and regional trends of people living with HIV aged 50 and over: estimates and projections for 2000–2020. PLoS One 2018; 13:1–11. 10.1371/journal.pone.0207005 PMC6264840 30496302

[2] Paula AA, Schechter M, Tuboi SH, et al Continuous increase of cardiovascular diseases, diabetes, and non-HIV related cancers as causes of death in HIV-infected individuals in Brazil: an analysis of nationwide data. PLoS One 2014; 9:1–5. 10.1371/journal.pone.0094636 PMC3984254 24728320

[3] Pathai S, Bajillan H, Landay AL, High KP. Is HIV a model of accelerated or accentuated aging? J Gerontol A Biol Sci Med Sci 2014; 69:833–42. 24158766 10.1093/gerona/glt168 PMC4067117

[4] López-Otín C, Blasco MA, Partridge L, Serrano M, Kroemer G. Hallmarks of aging: an expanding universe. Cell 2023; 186:243–78. 36599349 10.1016/j.cell.2022.11.001

[5] Ye Q, Apsley AT, Etzel L, et al Telomere length and chronological age across the human lifespan: a systematic review and meta-analysis of 414 study samples including 743,019 individuals. Ageing Res Rev 2023; 90:102031. 37567392 10.1016/j.arr.2023.102031 PMC10529491

[6] Arbeev KG, Verhulst S, Steenstrup T, et al Association of leukocyte telomere length with mortality among adult participants in 3 longitudinal studies. JAMA Netw Open 2020; 3:2–13. 10.1001/jamanetworkopen.2020.0023 PMC7137690 32101305

[7] Engel T, Raffenberg M, Schoepf IC, et al Telomere length, traditional risk factors, factors related to human immunodeficiency virus (HIV) and coronary artery disease events in Swiss persons living with HIV. Clin Infect Dis 2021; 73:e2070–6. 32725240 10.1093/cid/ciaa1034

[8] Gonzalez-Serna A, Ajaykumar A, Gadawski I, et al Rapid decrease in peripheral blood mononucleated cell telomere length after HIV seroconversion, but not HCV seroconversion. J Acquir Immune Defic Syndr 2017; 76:e29–32. 28797026 10.1097/QAI.0000000000001446 PMC6155455

[9] Jiménez VC, Wit FWNM, Joerink M, et al T-Cell activation independently associates with immune senescence in HIV-infected recipients of long-term antiretroviral treatment. J Infect Dis 2016; 214:216–25. 27073222 10.1093/infdis/jiw146 PMC8445638

[10] Schoepf IC, Thorball CW, Ledergerber B, et al Telomere length declines in persons with human immunodeficiency virus before antiretroviral therapy start but not after viral suppression: a longitudinal study over >17 years. J Infect Dis 2022; 225:1581–91. 34910812 10.1093/infdis/jiab603

[11] Montejano R, Stella-Ascariz N, Monge S, et al Impact of nucleos(t)ide reverse transcriptase inhibitors on blood telomere length changes in a prospective cohort of aviremic HIV-infected adults. J Infect Dis 2018; 218:1531–40. 29912427 10.1093/infdis/jiy364

[12] Comandini A, Naro C, Adamo R, et al Molecular mechanisms involved in HIV-1–Tat mediated inhibition of telomerase activity in human CD4+ T lymphocytes. Mol Immunol 2013; 54:181–92. 23287597 10.1016/j.molimm.2012.12.003

[13] Rodríguez-Centeno J, Esteban-Cantos A, Montejano R, et al Effects of tenofovir on telomeres, telomerase and T cell maturational subset distribution in long-term aviraemic HIV-infected adults. J Antimicrob Chemother 2022; 77:1125–32. 35045162 10.1093/jac/dkab492

[14] Montejano R, Stella-Ascariz N, Monge S, et al Impact of antiretroviral treatment containing tenofovir difumarate on the telomere length of aviremic HIV-infected patients. J Acquir Immune Defic Syndr 2017; 76:102–9. 28418989 10.1097/QAI.0000000000001391

[15] Rodríguez-Artalejo F, Graciani A, Guallar-Castillón P, et al Justificación y métodos del estudio sobre nutrición y riesgo cardiovascular en españa (ENRICA). Rev Esp Cardiol 2011; 64:876–82. 21821340 10.1016/j.recesp.2011.05.019

[16] Stella-Ascariz N, Montejano R, Rodriguez-Centeno J, et al Blood telomere length changes after ritonavir-boosted darunavir combined with raltegravir or tenofovir-emtricitabine in antiretroviral-naive adults infected with HIV-1. J Infect Dis 2018; 218:1523–30. 29982509 10.1093/infdis/jiy399

[17] Lombardi F, Sanfilippo A, Fabbiani M, et al Blood telomere length gain in people living with HIV switching to dolutegravir plus lamivudine versus continuing triple regimen: a longitudinal, prospective, matched, controlled study. J Antimicrob Chemother 2023; 78:2315–22. 37534393 10.1093/jac/dkad237 PMC10477130

[18] Leung JM, Fishbane N, Jones M, et al Longitudinal study of surrogate aging measures during human immunodeficiency virus seroconversion. Aging (Albany NY) 2017; 9:687–705. 28237978 10.18632/aging.101184 PMC5391226

[19] Zanet DAL, Thorne A, Singer J, et al Association between short leukocyte telomere length and HIV infection in a cohort study: no evidence of a relationship with antiretroviral therapy. Clin Infect Dis 2014; 58:1322–32. 24457340 10.1093/cid/ciu051

[20] Liu JCY, Leung JM, Ngan DA, et al Absolute leukocyte telomere length in HIV-infected and uninfected individuals: evidence of accelerated cell senescence in HIV-associated chronic obstructive pulmonary disease. PLoS One 2015; 10:1–13. 10.1371/journal.pone.0124426 PMC4401786 25885433

[21] Xia M, Liu K, Feng J, Zheng Z, Xie X. Prevalence and risk factors of type 2 diabetes and prediabetes among 53,288 middle-aged and elderly adults in China: a cross-sectional study. Diabetes Metab Syndr Obes 2021; 14:1975–85. 33976558 10.2147/DMSO.S305919 PMC8104985

[22] Cheng F, Carroll L, Joglekar MV, et al Diabetes, metabolic disease, and telomere length. Lancet Diabetes Endocrinol 2021; 9:117–26. 33248477 10.1016/S2213-8587(20)30365-X

[23] Bahour N, Cortez B, Pan H, Shah H, Doria A, Aguayo-Mazzucato C. Diabetes mellitus correlates with increased biological age as indicated by clinical biomarkers. GeroScience 2022; 44:415–27. 34773197 10.1007/s11357-021-00469-0 PMC8589453

[24] Park J, Zuñiga JA, García AA. Diabetes negatively impacts the ten-year survival rates of people living with HIV. Int J STD AIDS 2019; 30:991–8. 31335273 10.1177/0956462419857005 PMC7467551

[25] Schoepf IC, Esteban-Cantos A, Thorball CW, et al Epigenetic ageing accelerates before antiretroviral therapy and decelerates after viral suppression in people with HIV in Switzerland: a longitudinal study over 17 years. Lancet Healthy Longev 2023; 4:e211–8. 37148893 10.1016/S2666-7568(23)00037-5

[26] Gibellini L, Pecorini S, De Biasi S, et al HIV-DNA content in different CD4 + T-cell subsets correlates with CD4+ cell:CD8+ cell ratio or length of efficient treatment. AIDS 2017; 31:1387–92. 28426533 10.1097/QAD.0000000000001510

