# Supplementary material for: Partial Recovery of Telomere Length After Long-term Virologic Suppression in Persons With HIV-1
Source: Open Forum Infect Dis. 2024 Sep 23;11(10):ofae550. doi: 10.1093/ofid/ofae550 (PMC11482007; doi:10.1093/ofid/ofae550)
Supplement: ofae550_Supplementary_Data [file ofae550_supplementary_data.pdf]

Supplementary Material

Table of Contents

Supplementary Figures

- Supplementary Figure 1. Correlation between age and blood telomere length in the elderly population. **Page 2.**
- Supplementary Figure 2. Spaghetti plot of individual changes of blood telomere length in persons with HIV-1, at baseline and after six years of follow up. **Page 3.**
- Supplementary Figure 3. Comparison of blood telomere length in persons with HIV-1 with their age and sex-matched blood donors. Factors associated with similar blood telomere length (Univariate Analysis). **Page 4.**
- Supplementary Figure 4. Comparison of blood telomere length in Persons with HIV-1 with elderly general population over 60 years. Factors associated with similar blood telomere length (Univariate and multivariate analysis). **Page 5.**
- Supplementary Figure 5. Correlation between blood telomere length at baseline and after six years of follow-up in persons with HIV-1. **Page 6.**

Supplementary Tables

- Supplementary Table 1. Factors associated with blood telomere length (T/S) at six years of follow-up. **Page 7.**
- Supplementary Table 2. Factors associated with changes in blood telomere length (T/S) after six years of follow-up. **Page 9.**
- Supplementary Table 3. Factors associated with similarity of blood telomere length to healthy blood donors (within an established range of ±10%). **Page 11.**
- Supplementary Table 4. Factors associated with similarity of blood telomere length to the elderly population group (within an established range of ±10%). **Page 13.**

Supplementary Figure 1. Correlation between age and blood telomere length in the elderly population.

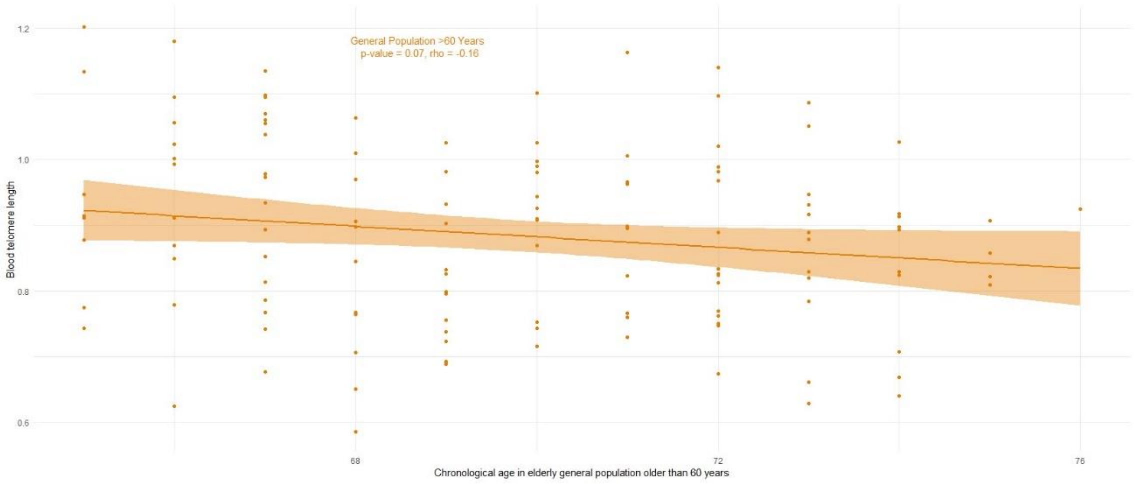

Correlation between age and blood telomere length (BTL) of the elderly population over 60 years group. Orange dots show individual data and the orange line the correlation curve. The shaded areas represent the 95% confidence interval.

Supplementary Figure 2. Spaghetti plot of individual changes of blood telomere length in persons with HIV-1, at baseline and after six years of follow up.

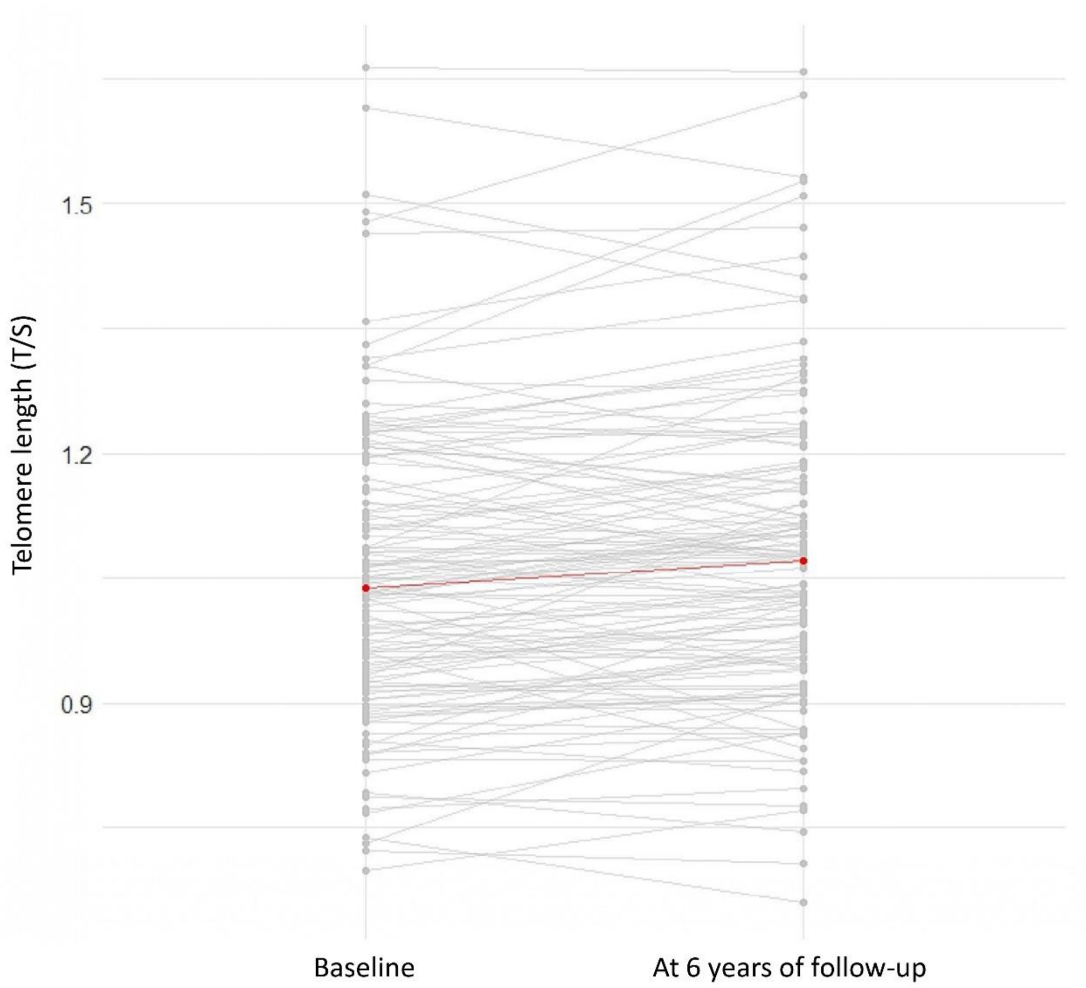

This spaghetti plot represents individual (gray lines) and median (red line) changes in blood telomere length (BTL) in people with HIV-1 (PWH) at baseline and 6-year follow-up.

Supplementary Figure 3. Comparison of blood telomere length in persons with HIV-1 with their age and sex-matched blood donors. Factors associated with similar blood telomere length (Univariate Analysis).

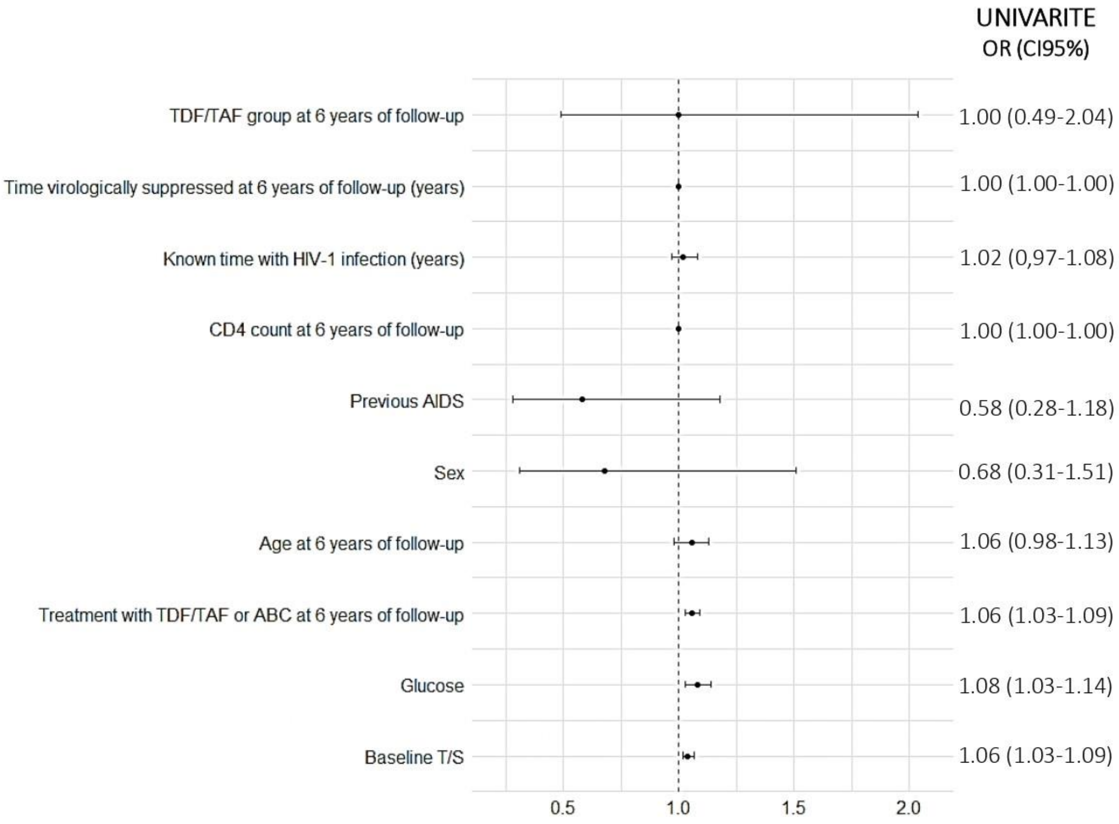

This forest plot shows the factors associated with the similarity of blood telomere length (BTL) of the people with HIV-1 (PWH) group with that of blood donors. For the comparison with blood donors “similar” refers to those PWH (median age 55 years), whose BTL falls within a range of ±10% or greater than the BTL observed in their age and sex-matched blood donor.

Supplementary Figure 4. Comparison of blood telomere length in Persons with HIV-1 with elderly general population over 60 years. Factors associated with similar blood telomere length (Univariate and multivariate analysis).

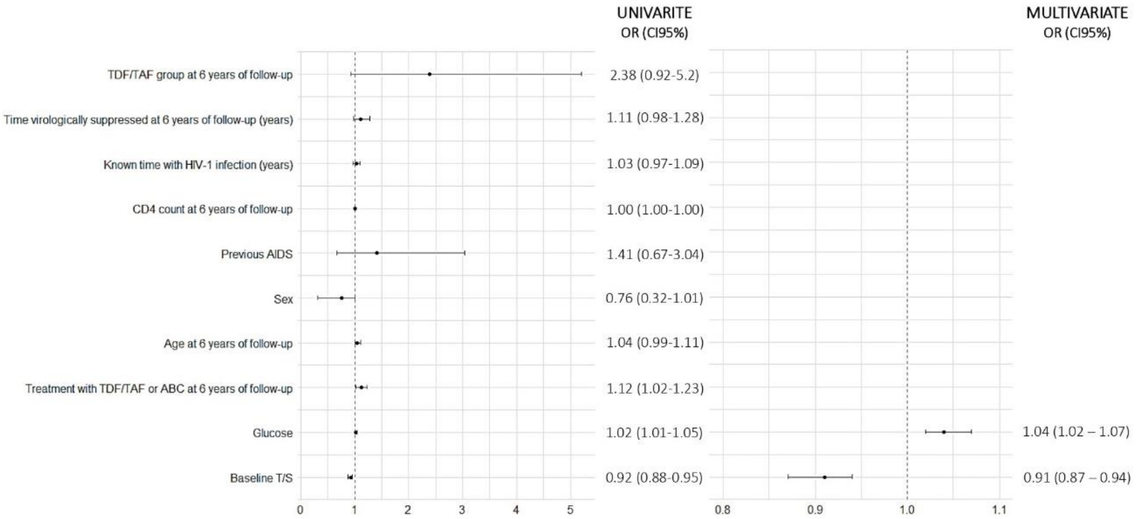

This forest plot shows the factors associated with the similarity of blood telomere length (BTL) of the people with HIV-1 (PWH) group with the elderly population group (over 60 years). For the comparison with the elderly population group "similar" refers to those PWH (median age 55 years), whose BTL falls within a range of ±10% or less than the median BTL observed in the elderly general population group (median age 70 years).

Supplementary Figure 5. Correlation between blood telomere length at baseline and after six years of follow-up in persons with HIV-1.

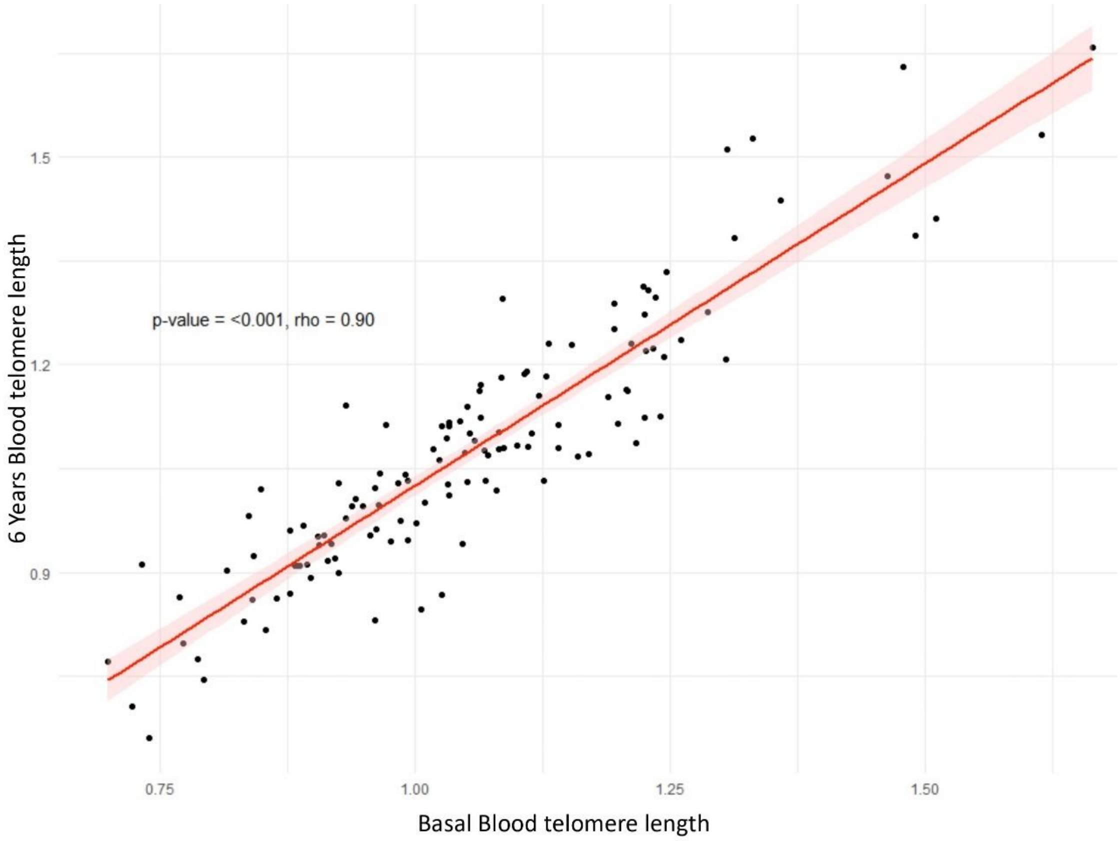

Correlation between blood telomere length (BTL) at baseline and after six years of follow-up in people with HIV-1 (PWH). Black dots show individual data and the red line the correlation curve. The shaded areas represent the 95% confidence interval.

Supplementary Table 1. Factors associated with blood telomere length (T/S) at six years of follow-up.

Variable	Coefficient	Std. Error	p
Baseline blood telomere length (T/S) x100	0·00957	0·00075	0·00000
Baseline Age	-0·00745	0·00221	0·00102
Change in blood telomere length (T/S) x100	0·00537	0·00321	0·09639
Age at 6 years of follow-up	-0·00745	0·00204	0·00204
Sex (Female)	0·05056	0·05922	0·39488
Ethnicity (caucasian)	-0·03369	0·05640	0·55138
Risk factor (parenteral)	-0·03173	0·18187	0·86179
Risk factor (sexual)	-0·02798	0·17931	0·87625
Income (≤ 12,000€/year)	0·05243	0·03686	0·15739
Previous AIDS	0·00846	0·03150	0·78877
Education (university)	-0·03575	0·04620	0·44049
Education (secondary)	-0·00339	0·03568	0·92451
Treatment with statins	-0·04584	0·03300	0·16727
Treatment with metformin	-0·04703	0·03944	0·23529
Active hepatitis C	-0·05931	0·03483	0·09104
Resolved hepatitis C	-0·01484	0·03490	0·67145
Body mass index	-0·00066	0·00425	0·87699
Smoker (current)	-0·02557	0·04691	0·58664
Smoker (formerly)	-0·07571	0·05209	0·14864
Smoker (current and formerly)	-0·03774	0·04754	0·42882
Smoker (years)	-0·00276	0·00170	0·10778
Smoker (pack years)	-0·00095	0·00053	0·07496
Alcohol (current)	-0·13106	0·07531	0·08427
Alcohol (formerly)	-0·00186	0·03447	0·95716
Alcohol (current and formerly)	-0·03609	0·06683	0·59017
Alcohol (years)	-0·00534	0·00555	0·36797
Alcohol (standard drink units)	-0·00273	0·00187	0·14841
Injected drug user	-	-	-
Baseline CD4 cell count	0·00006	0·00005	0·21689
Baseline CD4%	0·00230	0·00214	0·28539
Change in CD4 cell count	0·00001	0·00007	0·91029
Change in CD4%	-0·00012	0·00188	0·94989
CD4 cell count at 6 years of follow-up	0·00007	0·00007	0·30141
CD4% at 6 years of follow-up	-0·00012	0·00177	0·94545
CD8 cell count at 6 years of follow-up	0·00010	0·00006	0·10001
CD8% at 6 years of follow-up	-0·00093	0·00161	0·56632
CD4/CD8 ratio at 6 years of follow-up	-0·00273	0·01977	0·89036
C-Reactive protein	-0·00582	0·00585	0·32192
Glucose	-0·00048	0·00078	0·53877
Creatinine	0·00029	0·02419	0·99050
Total cholesterol	0·00054	0·00041	0·18994
HDL cholesterol	0·00193	0·00100	0·05529
LDL cholesterol	0·00025	0·00045	0·58269

Triglycerides	-0·00008	0·00027	0·76722
Fibrinogen	-0·00028	0·00025	0·26851
D-dimer	-0·00005	0·00011	0·61464
Positive CMV serology	-0·04793	0·04615	0·30098
Nadir CD4 cell count	-0·00004	0·00014	0·77189
Known time with HIV-1 infection (at 6 years of follow-up)	-0·00394	0·00251	0·11881
Time virologically suppressed (at 6 years of follow-up)	-0·00487	0·00778	0·53214
Group TDF/TAF at baseline (ever)	0·12322	0·07933	0·12286
Group TDF/TAF at baseline (ever+current)	-0·05753	0·03371	0·09029
Group TDF/TAF at 6 years of follow-up (Ever+current)	-0·05753	0·03371	0·09029
Group ABC at 6 years of follow-up (ever+current)	0·00425	0·03558	0·90512
Group NRTIs at 6 years of follow-up (ever+current)	-0·04784	0·03951	0·22821
Time with ABC (years)	-0·00519	0·00314	0·10257
Time with NRTIs (years)	-0·00519	0·00413	0·21083
Time with TDF/TAF (years)	-0·00183	0·00502	0·71744
Chronic kidney disease at baseline	-0·07658	0·11187	0·49491
Chronic kidney disease at 6 years follow-up	0·01961	0·06774	0·77268
Hypertension at baseline	-0·05714	0·03864	0·14173
Hypertension at 6 years follow-up	-0·01484	0·03487	0·67121
Diabetes mellitus at baseline	-0·04703	0·04368	0·28373
Diabetes mellitus at 6 years follow-up	-0·06084	0·03792	0·11110
Coronary artery disease at baseline	0·14893	0·07905	0·06187
Coronary artery disease at 6 years follow-up	0·11266	0·13911	0·41957
Peripheral Artery Disease at baseline	-	-	-
Peripheral Artery Disease at 6 years follow-up	-0·27524	0·09860	0·00606
1 comorbidity	0·00089	0·04084	0·98266
≥2 comorbidities	-0·06016	0·04420	0·17594

Supplementary Table 2. Factors associated with changes in blood telomere length (T/S) after six years of follow-up.

Variable	Coefficient	Std. Error	p
Baseline blood telomere length (T/S) x100	-0·00043	0·00061	0·48019
Baseline Age	0·00127	0·00161	0·43350
Blood telomere length at 6 years follow-up (T/S) x100	0·00134	0·00045	0·00342
Age at 6 years of follow-up	0·00127	0·00150	0·40031
Sex (Female)	-0·00001	0·02221	0·99982
Ethnicity (caucasian)	-0·03137	0·03938	0·42714
Risk factor (parenteral)	0·06876	0·04726	0·14817
Risk factor (sexual)	0·06875	0·04334	0·11520
Income (≤ 12,000€/year)	-0·00708	0·01912	0·71182
Previous AIDS	-0·00967	0·01983	0·62670
Education (university)	-0·00378	0·02003	0·85069
Education (secondary)	0·01593	0·02568	0·53619
Treatment with statins	-0·02257	0·02261	0·32016
Treatment with metformin	0·02261	0·03811	0·55412
Active hepatitis C	0·06608	0·04855	0·17596
Resolved hepatitis C	0·01667	0·02575	0·51850
Body mass index	-0·00012	0·00184	0·94940
Smoker (current)	-0·00398	0·03333	0·90521
Smoker (formerly)	0·00057	0·03224	0·98598
Smoker (current and formerly)	0·01523	0·03119	0·62615
Smoker (years)	-0·00033	0·00092	0·72297
Smoker (pack years)	-0·00048	0·00025	0·05769
Alcohol (current)	-0·00212	0·02375	0·92909
Alcohol (formerly)	0·06315	0·03787	0·09785
Alcohol (current and formerly)	-0·00212	0·02233	0·92458
Alcohol (years)	-0·00211	0·00192	0·30837
Alcohol (standard drink units)	-0·00029	0·00080	0·71947
Injected drug user	-	-	-
Baseline CD4 cell count	-0·00006	0·00002	0·00428
Baseline CD4%	-0·00180	0·00103	0·08089
Change in CD4 cell count	0·00005	0·00005	0·31355
Change in CD4%	-0·00009	0·00148	0·95392
CD4 cell count at 6 years of follow-up	-0·00005	0·00002	0·00217
CD4% at 6 years of follow-up	-0·00009	0·00108	0·93356
CD8 cell count at 6 years of follow-up	-0·00001	0·00004	0·89073
CD8% at 6 years of follow-up	0·00217	0·00128	0·09235
CD4/CD8 ratio at 6 years of follow-up	-0·00243	0·01593	0·87910
C-Reactive protein	-0·00158	0·00199	0·42671
Glucose	-0·00040		0·18965
Creatinine	0·00044	0·00753	0·95326
Total cholesterol	0·00000	0·00028	0·99982
HDL cholesterol	0·00112	0·00043	0·01114
LDL cholesterol	0·00000	0·00036	0·99977

Triglycerides	-0·00008	0·00012	0·47837
Fibrinogen	0·00002	0·00013	0·87496
D-dimer	0·00001	0·00004	0·82973
Positive CMV serology	0·03302	0·01713	0·05616
Nadir CD4 cell count	0·00008	0·00008	0·35211
Known time with HIV-1 infection (at 6 years of follow-up)	-0·00220	0·00158	0·16734
Time virologically suppressed (at 6 years of follow-up)	-0·00187	0·00365	0·60845
Group TDF/TAF at baseline (ever)	0·02551	0·02380	0·28583
Group TDF/TAF at baseline (ever+current)	-0·00001	0·01861	0·99978
Group TDF/TAF at 6 years of follow-up (Ever+current)	-0·00001	0·01861	0·99978
Group ABC at 6 years of follow-up (ever+current)	0·00001	0·01894	0·99979
Group NRTIs at 6 years of follow-up (ever+current)	-0·01052	0·06517	0·87204
Time with ABC (years)	-0·00353	0·00260	0·17827
Time with NRTIs (years)	0·00009	0·00231	0·96847
Time with TDF/TAF (years)	0·00234	0·00336	0·48917
Chronic kidney disease at baseline	0·03527	0·01936	0·07080
Chronic kidney disease at 6 years follow-up	0·03527	0·02160	0·10493
Hypertension at baseline	0·00001	0·01884	0·99978
Hypertension at 6 years follow-up	0·00001	0·01884	0·99978
Diabetes mellitus at baseline	0·00001	0·02529	0·99984
Diabetes mellitus at 6 years follow-up	0·00001	0·01869	0·99978
Coronary artery disease at baseline	-0·02953	0·01688	0·08262
Coronary artery disease at 6 years follow-up	-0·02953	0·05634	0·60116
Peripheral Artery Disease at baseline	-	-	-
Peripheral Artery Disease at 6 years follow-up	0·00144	0·03514	0·96739
1 comorbidity	-0·01537	0·02546	0·54720
≥2 comorbidities	0·00144	0·01972	0·94191

Supplementary Table 3. Factors associated with similarity of blood telomere length to healthy blood donors (within an established range of ±10%).

	Category	Overall	Worse	Similar or better	p
n		128	78	50	
Baseline blood telomere length (T/S, median [IQR])		1.04 [0.93, 1.16]	1·00 [0·91, 1·10]	1·10 [1·00, 1·24]	0·001
Change in blood telomere length at 6 years (T/S, median [IQR])		0.02 [-0.02, 0.08]	0·00 [-0·03, 0·06]	0·06 [-0·01, 0·08]	0·003
Blood telomere length at 6 years (T/S, median [IQR])		1.07 [0.95, 1.17]	1·02 [0·91, 1·10]	1·12 [1·03, 1·27]	<0·001
Sex (%)	Men	93 (72·7)	59 (75·6)	34 (68·0)	0·457
	Female	35 (27·3)	19 (24·4)	16 (32·0)	
Age at 6 years follow-up (years, median [IQR])		55·00 [51·00, 58·25]	53·50 [50·00, 58·00]	56·00 [52·00, 58·75]	0·060
Ethnicity (%	Caucasian	118 (92·2)	72 (92·3)	46 (92·0)	1·000
	Other	10 (7·8)	6 (7·7)	4 (8·0)	
Risk factor (%)	Unknown	4 (3·1)	3 (3·8)	1 (2·0)	0·802
	Parenteral	41 (32·0)	24 (30·8)	17 (34·0)	
	Sexual	83 (64·8)	51 (65·4)	32 (64·0)	
Income (%)	Higher (> 12,000 €/year)	78 (60·9)	49 (62·8)	29 (58·0)	0·719
	Lower (≤ 12,000 €/year)	50 (39·1)	29 (37·2)	21 (42·0)	
Previous AIDS	FALSE	56 (43·8)	30 (38·5)	26 (52·0)	0·186
	TRUE	72 (56·2)	48 (61·5)	24 (48·0)	
Treatment with statins (%)	FALSE	86 (67·2)	50 (64·1)	36 (72·0)	0·462
	TRUE	42 (32·8)	28 (35·9)	14 (28·0)	
Treatment with metformin (%)	FALSE	119 (93·0)	72 (92·3)	47 (94·0)	0·991
	TRUE	9 (7·0)	6 (7·7)	3 (6·0)	
Hepatitis C virus (%)	Active	2 (1·6)	2 (2·6)	0 (0·0)	0·510
	No	82 (64·1)	50 (64·1)	32 (64·0)	
	Resolved	44 (34·4)	26 (33·3)	18 (36·0)	
Smoking (%)	Current	59 (46·1)	32 (41·0)	27 (54·0)	0·179
	Formerly	41 (32·0)	25 (32·1)	16 (32·0)	
	Never	28 (21·9)	21 (26·9)	7 (14·0)	
Smoking combined (%)	Current and formerly	100 (78·1)	57 (73·1)	43 (86·0)	0·132
	Never	28 (21·9)	21 (26·9)	7 (14·0)	
Alcohol (%)	Current	7 (5·5)	5 (6·4)	2 (4·0)	0·178
	Formerly	2 (1·6)	0 (0·0)	2 (4·0)	
	Never	119 (93·0)	73 (93·6)	46 (92·0)	
Alcohol combined (%)	Current and formerly	9 (7·0)	5 (6·4)	4 (8·0)	1000
	Never	119 (93·0)	73 (93·6)	46 (92·0)	
Baseline CD4 cell count (cells/µL, median [IQR])		769·15 [580·28, 1,040·25]	785·20 [546·80, 1,027·65]	731·20 [585·00, 1,039·65]	0·971
Baseline CD4% (median [IQR])		37·00 [29·75, 42·25]	37·00 [31·00, 40·75]	37·00 [29·00, 42·75]	0·841
CD4 cell count at 6 years of follow-up (cells/µL, median [IQR])		773.50 [540.00, 1,020.00]	710·00 [535·00, 980·00]	780·00 [570·00, 1,050·00]	0·623
CD4% at 6 years of follow-up (median [IQR])		37.00 [31.00, 42.61]	37·00 [31·00, 42·75]	36·00 [32·00, 42·61]	0·940

Change in CD4 cell count (cells/µL, median [IQR])		-4·80 [-157·78, 96·60]	-65·00 [-179·30, 82·40]	9·00 [-148·00, 146·60]	0·330
Change in CD4% (cells/µL, median [IQR])		1·00 [-2·50, 5·00]	1·00 [-2·25, 4·00]	3·00 [-2·50, 6·00]	0·123
CD8 cell count at 6 years of follow-up (cells/µL, median [IQR])		674.00 [470.00, 930.00]	675·00 [462·50, 895·00]	674·00 [495·00, 985·00]	0·541
CD8% at 6 years of follow-up (median [IQR])		33.00 [29.00, 40.00]	34·00 [29·50, 40·00]	33·00 [28·50, 40·50]	0·823
CD4/CD8 ratio at 6 years of follow-up (median [IQR])		1.11 [0.80, 1.44]	1·09 [0·79, 1·44]	1·15 [0·81, 1·42]	0·987
C-Reactive protein (mg/l, median [IQR])		1·30 [0·00, 4·27]	1·40 [0·00, 4·70]	0·80 [-0·00, 3·10]	0·343
Glucose (mg/dl, median [IQR])		91·00 [82·75, 101·00]	91·00 [82·25, 100·50]	91·00 [84·00, 100·75]	0·044
Creatinine (mg/dl, median [IQR])		0·85 [0·75, 0·93]	0·84 [0·74, 0·95]	0·85 [0·75, 0·92]	0·760
Cholesterol (mg/dl, median [IQR])		181·00 [152·00, 204·25]	177·00 [148·00, 205·75]	182·00 [161·25, 204·00]	0·596
HDL cholesterol (mg/dl, median [IQR])		48·50 [39·00, 58·00]	45·50 [37·25, 56·75]	52·00 [43·25, 58·75]	0·062
LDL cholesterol (mg/dl, median [IQR])		108·00 [85·00, 131·00]	108·00 [82·75, 132·75]	108·00 [86·00, 129·25]	0·721
Triglycerides (mg/dl, median [IQR])		97·50 [75·75, 151·25]	102·50 [76·25, 158·25]	87·50 [74·50, 127·50]	0·283
Fibrinogen (mg/dl, median [IQR])		308·00 [260·00, 372·75]	302·00 [247·50, 374·00]	318·00 [272·50, 360·50]	0·572
D-Dimer (ng/ml, median [IQR])		220·00 [170·00, 312·50]	210·00 [170·00, 335·00]	220·00 [170·00, 290·00]	0·638
Positive CMV serology (%)	NO	12 (9·4)	9 (11·5)	3 (6·0)	0·460
	YES	116 (90·6)	69 (88·5)	47 (94·0)	
Nadir CD4 count (cells/µL, median [IQR])		181·50 [80·50, 255·00]	182·50 [82·50, 258·25]	180·50 [79·50, 248·75]	0·764
Known time with HIV-1 infection (years, median [IQR])		23.58 [18.46, 27.93]	23·33 [18·48, 27·75]	23·95 [18·67, 28·36]	0·600
Time virologically suppressed (at 6 years of follow-up) (years, median [IQR])		13.23 [12.07, 13.67]	13·15 [11·03, 13·62]	13·47 [12·59, 13·75]	0·248
Comorbidities (%)	1	29 (22·7)	15 (19·2)	14 (28·0)	0·482
	2 or more	24 (18·8)	16 (20·5)	8 (16·0)	
	No	75 (58·6)	47 (60·3)	28 (56·0)	
Group TDF/TAF at 6 years of follow-up (%)	Ever + Actual	64 (50·0)	39 (50·0)	25 (50·0)	1·000
	Never	64 (50·0)	39 (50·0)	25 (50·0)	
Group ABC at 6 years of follow-up (%)	Ever + Actual	76 (59·4)	48 (61·5)	28 (56·0)	0·661
	Never	52 (40·6)	30 (38·5)	22 (44·0)	
Group NRTI at 6 years of follow-up (%)	Ever + Actual	120 (93·8)	76 (97·4)	44 (88·0)	0·005
	Never	8 (6·2)	2 (2·6)	6 (12·0)	
Time with ABC (years, median [IQR])		9·83 [5·14, 12·91]	10·31 [8·03, 13·21]	6·96 [4·10, 12·91]	0·154
Time with NRTIs (years, median [IQR])		14·33 [9·96, 16·82]	14·46 [9·96, 16·79]	14·02 [10·67, 16·91]	0·845
Time with TDF/TAF (years, median [IQR])		15·47 [11·52, 16·93]	15·60 [11·40, 16·95]	14·39 [11·52, 16·84]	0·788

Supplementary Table 4. Factors associated with similarity of blood telomere length to the elderly population group (within an established range of ±10%).

	Category	Overall	Similar or worse	Better	p
n		128	42	86	
Baseline blood telomere length (T/S, median [IQR])		1.04 [0.93, 1.16]	0·93 [0·84, 1·00]	1·09 [1·01, 1·22]	<0·001
Change in blood telomere length (T/S, median [IQR])		0.02 [-0.02, 0.08]	0·02 [-0·02, 0·06]	0·03 [-0·02, 0·08]	0·429
Blood telomere length at 6 years (T/S, median [IQR])		1.07 [0.95, 1.17]	0·95 [0·89, 1·03]	1·11 [1·03, 1·23]	<0·001
Sex (%)	Men	93 (72·7)	32 (76·2)	61 (70·9)	0·678
	Female	35 (27·3)	10 (23·8)	25 (29·1)	
Age at 6 years follow-up (years, median [IQR])		55·00 [51·00, 58·25]	55·00 [51·00, 59·00]	54·50 [50·00, 57·00]	0·364
Ethnicity (%)	Caucasian	118 (92·2)	39 (92·9)	79 (91·9)	1·000
	Other	10 (7·8)	3 (7·1)	7 (8·1)	
Risk factor (%)	Unknown	4 (3·1)	1 (2·4)	3 (3·5)	0·929
	Parenteral	41 (32·0)	14 (33·3)	27 (31·4)	
	Sexual	83 (64·8)	27 (64·3)	56 (65·1)	
Income (%)	Higher (> 12,000 €/year)	78 (60·9)	25 (59·5)	53 (61·6)	0·971
	Lower (≤ 12,000 €/year)	50 (39·1)	17 (40·5)	33 (38·4)	
Previous AIDS (%)	FALSE	56 (43·8)	16 (38·1)	40 (46·5)	0·477
	TRUE	72 (56·2)	26 (61·9)	46 (53·5)	
Treatment with statins (%)	FALSE	86 (67·2)	27 (64·3)	59 (68·6)	0·773
	TRUE	42 (32·8)	15 (35·7)	27 (31·4)	
Treatment with metformin (%)	FALSE	119 (93·0)	38 (90·5)	81 (94·2)	0·687
	TRUE	9 (7·0)	4 (9·5)	5 (5·8)	
Hepatitis C virus (%)	Active	2 (1·6)	2 (4·8)	0 (0·0)	0·125
	No	82 (64·1)	26 (61·9)	56 (65·1)	
	Resolved	44 (34·4)	14 (33·3)	30 (34·9)	
Smoking (%)	Current	59 (46·1)	15 (35·7)	44 (51·2)	0·222
	Formerly	41 (32·0)	15 (35·7)	26 (30·2)	
	Never	28 (21·9)	12 (28·6)	16 (18·6)	
Smoking combined (%)	Current and formerly	100 (78·1)	30 (71·4)	70 (81·4)	0·292
	Never	28 (21·9)	12 (28·6)	16 (18·6)	
Alcohol (%)	Current	7 (5·5)	1 (2·4)	6 (7·0)	0·331
	Formerly	2 (1·6)	0 (0·0)	2 (2·3)	
	Never	119 (93·0)	41 (97·6)	78 (90·7)	
Alcohol combined (%)	Current and formerly	9 (7·0)	1 (2·4)	8 (9·3)	0·285
	Never	119 (93·0)	41 (97·6)	78 (90·7)	
Baseline CD4 cell count (cells/µL, median [IQR])		769·15 [580·28, 1,040·25]	685·10 [486·38, 1,004·05]	795·90 [599·20, 1,045·95]	0·238
Baseline CD4% (median [IQR])		37·00 [29·75, 42·25]	35·00 [29·00, 40·00]	38·00 [30·00, 43·00]	0·132
CD4 cell count at 6 years of follow-up (cells/µL, median [IQR])		773.50 [540.00, 1,020.00]	670·00 [528·50, 914·00]	785·00 [560·00, 1,035·00]	0·224
CD4% at 6 years of follow-up (median [IQR])		37.00 [31.00, 42.61]	35·50 [30·00, 39·50]	38·00 [31·00, 45·00]	0·307

Change in CD4 cell count (cells/µL, median [IQR])		-4·80 [-157·78, 96·60]	27·60 [-127·83, 75·57]	-6·50 [-185·65, 117·25]	0·605
Change in CD4% (median [IQR])		1·00 [-2·50, 5·00]	1·00 [-2·00, 5·50]	2·00 [-3·00, 5·00]	0·996
CD8 cell count at 6 years of follow-up (cells/µL, median [IQR])		674.00 [470.00, 930.00]	650·00 [460·00, 836·25]	674·00 [495·00, 950·00]	0·310
CD8% at 6 years of follow-up (median [IQR])		33.00 [29.00, 40.00]	33·00 [30·00, 39·75]	33·50 [28·00, 40·25]	0·857
CD4/CD8 ratio at 6 years of follow-up (median [IQR])		1.11 [0.80, 1.44]	1·05 [0·80, 1·31]	1·15 [0·80, 1·47]	0·620
C-Reactive protein (mg/l, median [IQR])		1·30 [0·00, 4·27]	0·80 [0·00, 3·00]	1·40 [0·00, 4·40]	0·212
Glucose (mg/dl, median [IQR])		91·00 [82·75, 101·00]	95·50 [88·00, 107·50]	88·50 [81·00, 96·00]	0·001
Creatinine (mg/dl, median [IQR])		0·85 [0·75, 0·93]	0·86 [0·80, 0·94]	0·84 [0·73, 0·93]	0·443
Total cholesterol (mg/dl, median [IQR])		181·00 [152·00, 204·25]	182·50 [152·00, 205·75]	180·50 [154·00, 204·00]	0·957
HDL cholesterol (mg/dl, median [IQR])		48·50 [39·00, 58·00]	47·50 [37·25, 58·00]	48·50 [40·00, 58·75]	0·490
LDL cholesterol (mg/dl, median [IQR])		108·00 [85·00, 131·00]	104·50 [86·25, 131·75]	109·00 [82·25, 130·75]	0·755
Triglycerides (mg/dl, median [IQR])		97·50 [75·75, 151·25]	99·00 [75·25, 162·75]	97·50 [78·00, 138·50]	0·815
Fibrinogen (mg/dl, median [IQR])		308·00 [260·00, 372·75]	293·00 [244·00, 373·50]	317·00 [269·50, 367·50]	0·241
D-Dimer (ng/ml, median [IQR])		220·00 [170·00, 312·50]	192·00 [170·00, 270·00]	230·00 [170·00, 345·00]	0·195
Positive CMV serology (%)	NO	12 (9·4)	5 (11·9)	7 (8·1)	0·716
	YES	116 (90·6)	37 (88·1)	79 (91·9)	
Nadir CD4 count (cells/µL, median [IQR])		181·50 [80·50, 255·00]	139·00 [37·25, 244·75]	186·50 [102·00, 255·00]	0·230
Known time with HIV-1 infection (years, median [IQR])		23.58 [18.46, 27.93]	22·84 [19·73, 28·85]	23·82 [17·60, 27·87]	0·476
Time virologically suppressed (at 6 years of follow-up) (years, median [IQR])		13.23 [12.07, 13.67]	13·30 [12·80, 13·56]	13·12 [10·59, 13·68]	0·321
Comorbidities (%)	1	29 (22·7)	7 (16·7)	22 (25·6)	0·510
	2 or more	24 (18·8)	9 (21·4)	15 (17·4)	
	No	75 (58·6)	26 (61·9)	49 (57·0)	
Group TDF/TAF at 6 years of follow-up (%)	Ever + Actual	64 (50·0)	27 (64·3)	37 (43·0)	0·038
	Never	64 (50·0)	15 (35·7)	49 (57·0)	
Group ABC at 6 years of follow-up (%)	Ever + Actual	76 (59·4)	23 (54·8)	53 (61·6)	0·582
	Never	52 (40·6)	19 (45·2)	33 (38·4)	
Group NRTI at 6 years of follow-up (%)	Ever + Actual	120 (93·8)	42 (100·0)	78 (90·7)	0·098
	Never	8 (6·2)	0 (0·0)	8 (9·3)	
Time with ABC (years, median [IQR])		9·83 [5·14, 12·91]	10·32 [6·81, 14·31]	9·77 [4·88, 12·88]	0·456
Time with NRTIs (years, median [IQR])		14·33 [9·96, 16·82]	15·91 [11·20, 17·11]	12·97 [9·78, 15·87]	0·016
Time with TDF/TAF (years, median [IQR])		15·47 [11·52, 16·93]	16·08 [12·77, 17·10]	13·57 [11·49, 16·81]	0·148
